# From the Cochlea to the Cortex: Toward Integrated Auditory Health—Editorial for the Special Issue “*Recent Advances in Hearing Impairment*”

**DOI:** 10.3390/brainsci16060640

**Published:** 2026-06-16

**Authors:** Agnieszka J. Szczepek

**Affiliations:** 1 Department of Otorhinolaryngology, Head and Neck Surgery, Charité-Universitätsmedizin Berlin, Corporate Member of Freie Universität Berlin and Humboldt-Universität zu Berlin, 10117 Berlin, Germany; agnes.szczepek@charite.de; 2 Faculty of Medicine and Health Sciences, University of Zielona Góra, 65-046 Zielona Góra, Poland

## 1. Introduction

**Figure 1:**
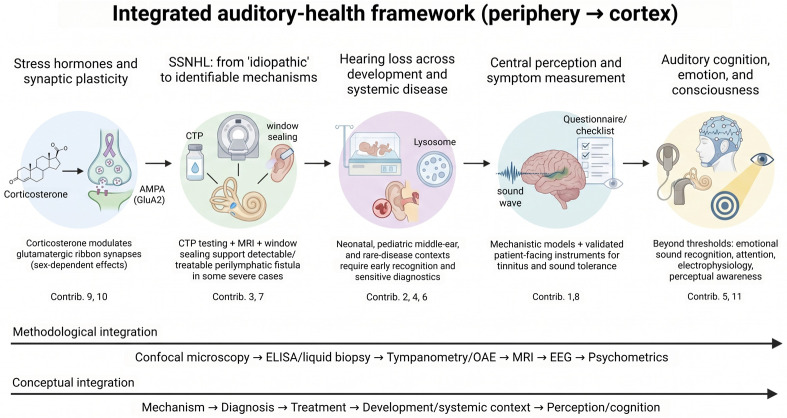
Integrated auditory health framework for the Special Issue “Recent Advances in Hearing Impairment.” The eleven contributions exist along a continuum from peripheral molecular and synaptic mechanisms to clinical diagnosis; developmental and systemic disease contexts; tinnitus and sound-tolerance assessment; and cortical mechanisms of emotion, cognition, attention, and awareness. The lower arrows summarize the methodological integration across studies and the conceptual shift from organ-centered hearing research toward integrated auditory health. Created with BioRender.com.

Hearing impairment is among the most prevalent sensory disorders worldwide, affecting far more than just auditory thresholds. It changes how the brain interprets sound, impacting communication, emotion, and cognition, and often signals broader systemic or developmental problems. Progress in this area depends on coordinated efforts across multiple levels: from understanding molecular mechanisms at the synapse, through clinical diagnosis and treatments, to the cortical networks responsible for perception and consciousness. This Special Issue, “Recent Advances in Hearing Impairment,” compiles eleven contributions that follow the auditory pathway: from the periphery to the cortex. It features original experimental and clinical research, as well as systematic and scoping reviews. Figure 1 summarizes this Special Issue as a framework for auditory health, presenting eleven contributions from molecular mechanisms in the auditory periphery to cortical processing, emotion, cognition, and awareness.

## 2. Molecular and Synaptic Mechanisms: Stress Hormones and the Auditory Pathway

Two contributions from the same research group explored a surprisingly understudied question: how stress hormones influence glutamatergic synapses involved in hearing. AMPA-type ionotropic glutamate receptors facilitate rapid excitatory signaling across the central nervous system and are crucial for auditory processing. **Edlund et al**. (**Contribution 9**) present a scoping review on how corticosterone, the main stress hormone in rodents, affects AMPA receptor levels and positioning in the rodent brain. After screening 288 articles and narrowing down to 17, they found no studies specifically focused on the auditory system. However, they provide a clear overview from neuronal cultures, showing that short-term corticosterone exposure increases AMPA receptor surface localization and mobility, especially in the hippocampus and prefrontal cortex. They also highlight significant inconsistencies among whole-animal studies and strongly advocate for standardized methodologies. **Domarecka et al**. (**Contribution 10**) investigate the cochlea directly. Using cochlear explants from male and female C57BL/6 mouse pups, they demonstrate that a brief corticosterone pulse alters the size and synaptic placement of Ribeye and the GluR2 subunit at the inner hair cell ribbon synapse, the first afferent synapse of the auditory pathway, and that these changes are sex-dependent. Their finding that corticosterone induces neuroplasticity-like changes in the auditory periphery fill a gap identified in the review and emphasize the importance of considering sex as a biological factor in hearing research.

## 3. Sudden Sensorineural Hearing Loss: Perilymphatic Fistula, Biomarkers, and First-Line Management

Two small prospective case series examined the diagnosis and management of severe sudden sensorineural hearing loss (SSNHL), focusing on cochlin-tomoprotein (CTP), an inner-ear protein used as a biomarker of perilymph leakage and suspected perilymphatic fistula (PLF). This raises the question of whether some cases traditionally labeled as “idiopathic” SSNHL may in fact have identifiable mechanisms. **Kim et al.** (**Contribution 3**) investigated the relationship between positive CTP testing, MRI findings, and auditory and vestibular functions in patients with sudden hearing loss. They used delayed gadolinium-enhanced 3D-FLAIR imaging along with ELISA-based CTP detection in middle ear lavage. Their results showed non-specific cochlear and vestibular contrast enhancement on the affected side in all patients and positive CTP in half of the patients, but no MRI signals specific to PLF, highlighting the limitations of current multimodal diagnostic methods. **Kilgue et al.** (**Contribution 7**) assessed CTP detection alongside round- and oval-window sealing as an initial surgical approach for severe SSNHL. In a study of 30 patients, bone-conduction thresholds generally improved after middle ear exploration and window sealing. In 21 patients (70%), the CTP concentration in middle ear lavage exceeded 60 ng/mL; in 8 (27%), it was between 30 and 60 ng/mL; and in only 1 case was it negative. Together, these findings support further investigation into whether some cases of severe idiopathic sudden sensorineural hearing loss may be explained by occult perilymphatic fistulas that are biomarker-detectable and potentially treatable. Although these studies are still exploratory, they highlight a broader trend in otology: a shift from relying solely on idiopathic labels to adopting diagnostic methods grounded in biomarkers and underlying mechanisms.

## 4. Hearing Loss Across Development and in Systemic Disease

Three contributions expand hearing research by focusing on specific populations, highlighting early detection, comorbidities, and the otolaryngologist’s role in conditions in which hearing loss is part of a broader clinical picture. **Moosan et al.** (**Contribution 4**) conducted a retrospective, case–control study of infants admitted to the neonatal intensive care unit (NICU). They examined how prematurity correlates with permanent childhood hearing impairment (PCHI) and neurodevelopmental outcomes at 2 years of age. The study found that both PCHI and NICU admission independently predicted poor development, regardless of the severity of prematurity. Additionally, the number of days spent in the NICU, rather than birth-weight z-scores, was a predictor of PCHI. In practice, these results indicate that NICU history, length of stay, and early hearing status should be included in developmental follow-up plans, rather than relying solely on prematurity or birth weight to identify at-risk infants. Early detection of PCHI should lead to increased neurodevelopmental monitoring and earlier intervention. **Sanches et al.** (**Contribution 2**) investigated middle ear function in children after surgical removal of the palatine and/or pharyngeal tonsils, using wideband tympanometry and pressurized otoacoustic emissions. Their research highlights the importance of more sensitive, frequency-specific methods for assessing middle ear health in a common pediatric surgical group. **Waśniewska-Włodarczyk et al.** (**Contribution 6**) examined the otorhinolaryngological symptoms of mucopolysaccharidoses (MPS), a rare group of lysosomal storage diseases in which most patients experience otitis media, craniofacial abnormalities, obstructive sleep apnea, and hearing impairment. This narrative review warns clinicians that, in children, recurrent ENT disease plus craniofacial, airway, hearing, developmental, or skeletal abnormalities should prompt suspicion of MPS, because early recognition by ENT doctors or pediatricians may accelerate diagnosis, enable earlier systemic treatment, and reduce preventable airway- and hearing-related morbidity.

Together, these studies remind clinicians that hearing impairment is often not an isolated sensory deficit, but rather an early marker of developmental, systemic, or syndromic vulnerability.

## 5. Tinnitus and Disorders of Sound Tolerance

Two studies address perceptual disorders in which central processing, sensory prediction, attention, and affective response play important roles: tinnitus and decreased sound tolerance. **Shahin et al.** (**Contribution 1**) present a theoretical model for cross-modal tinnitus treatment. They build on the idea that tinnitus typically results from deafferentation, the loss of high-frequency input from the cochlea, leading to abnormal synchronization in cortical neurons. They suggest that consistent audiovisual engagement, leveraging vision’s influence on auditory pathways, could gradually reorganize these neural groups and lessen tinnitus severity over time. This framework provides a mechanism-based, testable basis for multisensory intervention. **Aazh and Kula** (**Contribution 8**) focus on measuring sound-tolerance disorders by validating the Sound Sensitivity Symptoms Questionnaire (SSSQ2) version 2. In a large sample, this six-item instrument proved to be a reliable single-factor measure of overall sound sensitivity, demonstrating good test–retest reliability. Its individual items can function as a screening checklist for loudness and pain hyperacusis, misophonia, fear hyperacusis, and noise sensitivity. The reported minimum detectable change provides valuable thresholds for clinicians and researchers to interpret score variations. Brief, validated tools like this are crucial for accurately identifying conditions that overlap under the umbrella of reduced sound tolerance.

## 6. The Hearing Brain: Cognition, Emotion, and Electrophysiology

Finally, two studies explored how the brain processes sound in contexts related to hearing impairment and attention. In their exploratory study, **Cartocci et al.** (**Contribution 5**) examined whether adult cochlear implant users have difficulty recognizing emotional content in sounds by combining EEG measurements of gamma and alpha asymmetry with an assessment of alexithymia, defined as difficulty identifying and describing one’s own emotions. They found that adult unilateral CI users have a measurable deficit in their recognition of musical emotions, particularly for sad stimuli, and that this deficit is accompanied by altered gamma-band activity. Their approach links electrophysiological asymmetry to emotion-recognition ability, highlighting an aspect of cochlear implant outcomes not fully captured by standard audiometric measures. **Alain et al**. (**Contribution 11**) investigated neural markers of perceptual awareness during the auditory attentional blink by combining data from six prior studies to increase statistical power. They found that correctly reporting both targets was linked to increased frontocentral negativity shortly after the second target, a subsequent central–parietal positivity, and stronger alpha suppression. Additionally, higher pre-stimulus alpha power may indicate attentional lapses. This research enhances our understanding of the electrophysiological signals associated with auditory awareness and highlights the influence of ongoing brain states on what enters conscious perception.

## 7. Conclusions and Outlook

This Special Issue features eleven contributions that highlight the extensive scope of modern hearing research and the importance of integrating multiple analysis levels. Several key themes are evident. First, the connection between basic research and clinical practice is increasingly vital, demonstrated by studies linking molecular and synaptic mechanisms to clinically relevant issues such as sudden sensorineural hearing loss, cochlear implantation, tinnitus, and sound tolerance. Second, understanding hearing disorders requires more than just examining the ear; neural reorganization, emotional sound processing, auditory attention, and perceptual awareness all emphasize the brain’s central role in auditory health. Third, some contributions point to methodological priorities, including standardized protocols; sex as a biological variable; multimodal diagnostics; and the implementation of validated, sensitive patient-reported and electrophysiological measures.

Many studies in this Special Issue are exploratory or hypothesis-generating, with several findings needing validation through larger, longitudinal, and multicenter studies. Nonetheless, they highlight methodological and conceptual paths likely to influence future auditory research. Three main messages emerge clearly: First, understanding auditory disorders requires mechanistic explanations spanning from synaptic plasticity to cortical awareness. Second, advancing clinical progress involves integrating imaging, biomarkers, electrophysiology, psychometrics, and patient-centered outcomes. Third, hearing impairment should be viewed within developmental, systemic, emotional, and cognitive contexts, not merely as a deficit in auditory thresholds. As shown in Figure 1, this Special Issue signifies a transition from organ-centered hearing research to a more holistic view of auditory health.

I thank all the authors for their contributions, the reviewers for their thorough and constructive feedback, and the *Brain Sciences* editorial team for their support. I hope this collection will prove useful to researchers and clinicians alike and will encourage the interdisciplinary collaboration that hearing impairment naturally requires.

